# Corneal biomechanics in normal and subclinical keratoconus eyes

**DOI:** 10.1186/s12886-023-03215-6

**Published:** 2023-11-15

**Authors:** Alireza Peyman, Fatemeh Sepahvand, Mohsen Pourazizi, Pegah Noorshargh, Ali Forouhari

**Affiliations:** 1 Isfahan Eye Research Center, Department of Ophthalmology, Isfahan University of Medical Sciences, Isfahan, Iran; 2 School of Medicine, Isfahan University of Medical Sciences, Isfahan, Iran

**Keywords:** Subclinical keratoconus, Corvis ST, Scheimpflug technology, Corneal biomechanics

## Abstract

**Background:**

The diagnosis of keratoconus, as the most prevalent corneal ectatic disorder, at the subclinical stage gained great attention due to the increased acceptance of refractive surgeries. This study aimed to assess the pattern of the corneal biomechanical properties derived from Corneal Visualization Scheimpflug Technology (Corvis ST) and evaluate the diagnostic value of these parameters in distinguishing subclinical keratoconus (SKC) from normal eyes.

**Methods:**

This prospective study was conducted on 73 SKC and 69 normal eyes. Subclinical keratoconus eyes were defined as corneas with no clinical evidence of keratoconus and suspicious topographic and tomographic features. Following a complete ophthalmic examination, topographic and tomographic corneal assessment via Pentacam HR, and corneal biomechanical evaluation utilizing Corvis ST were done.

**Results:**

Subclinical keratoconus eyes presented significantly higher Deformation Amplitude (DA) ratio, Tomographic Biomechanical Index (TBI), and Corvis Biomechanical Index (CBI) rates than the control group. Conversely, Ambrósio Relational Thickness to the Horizontal profile (ARTh), and Stiffness Parameter at the first Applanation (SPA1) showed significantly lower rates in SKC eyes. In diagnosing SKC from normal eyes, TBI (AUC: 0.858, Cut-off value: > 0.33, Youden index: 0.55), ARTh (AUC: 0.813, Cut-off value: ≤ 488.1, Youden index: 0.58), and CBI (AUC: 0.804, Cut-off value: > 0.47, Youden index: 0.49) appeared as good indicators.

**Conclusions:**

TBI, CBI, and ARTh parameters could be valuable in distinguishing SKC eyes from normal ones.

## Introduction

Keratoconus is a bilateral non-symmetrical ectatic corneal dystrophy [1, 2]. This degenerative ocular disease usually arises in the second and third decade of life and could lead to irregular astigmatism and loss of visual acuity [1, 3]. Keratoconus detection at the early stages would result in a better long-term prognosis, and it is crucial to be ruled out before refractive surgeries [1, 4]. Performing refractive surgeries on keratoconus suspect eyes could lead to postoperative iatrogenic progressive ectatic disorder of the cornea [5]. Due to the alteration of corneal biomechanical and viscoelastic properties in keratoconus pathophysiology [6], it has been proposed that corneal biomechanical parameters could detect subclinical keratoconus patients before the appearance of significant changes in the corneal topography and tomography [2, 7].

The Corneal Visualization Scheimpflug Technology (Corvis ST) is a relatively new non-contact device that presents corneal biomechanical properties through dynamic imaging assessment of the corneal deformation in response to the applied external force [8, 9]. Numerous clinical studies evaluated the diagnostic value and the discriminatory potential of the corneal biomechanical parameters, provided via the Corvis ST, in distinguishing keratoconus suspect eyes from normal ones [5, 10, 11]. Although a few studies mentioned that the existing corneal biomechanics parameters, determined by Corvis ST, are not sensitive and reliable metrics to discriminate subclinical keratoconus from normal eyes in isolation [1, 12], various clinical studies showed the diagnostic value of those parameters in preliminary keratoconus [13, 14]. However, the definitions and diagnostic criteria used for subclinical keratoconus and the reported accuracy of the Corvis ST parameters vary greatly.

This study aimed to compare the biomechanical properties between keratoconus suspect -normal appearing corneas with suspicious topographic and tomographic features- and normal corneas, and assess the value of these parameters in the diagnosis of subclinical keratoconus.

## Materials and methods

This prospective observational study was conducted at the PARSIAN eye clinic and research center, and Feiz Hospital, Isfahan University of Medical Sciences. The research was approved by the Institutional Review Board of the Isfahan University of Medical Sciences (project number: 399043, ethical approval ID: IR.MUI.MED.REC.1399.131). Candidates referred to the hospital for refractive surgeries between August 2021 and May 2022 underwent a comprehensive ophthalmic examination. This study followed the tenets of the Declaration of Helsinki, and all the included participants signed written informed consent. Keratoconus suspect cornea was defined as 1- Normal-appearing on slit-lamp bio-microscopy, retinoscopy, ophthalmoscopy, and keratometry [15, 16]. 2- Inferior-superior asymmetry or bow-tie pattern with skewed radial axes (suspicious topographic findings) showed in tangential maps of the Pentacam [15, 16] 3- Belin/Ambrósio enhanced ectasia total deviation value (BAD-D index) score, revealed by Pentacam HR, between 1.6 and 3.0 standard deviations (SDs) from normative rates [17]. Eyes with normal topographic features and a BAD-D score of less than 1.6, which showed no clinical evidence of keratoconus, were recruited as the control group. In the case that a participant had one normal eye and subclinical keratoconus in the fellow eye, the keratoconus suspect eye was included. If both eyes of each subject had a similar state, one was randomly selected. Exclusion criteria were as the following: previous ocular surgery or trauma, any previous or concomitant corneal pathology (e.g., corneal scar or history of corneal hydrops, glaucoma or hypotonic therapies) or other ocular diseases, wearing soft and rigid contact lenses within four weeks before the examination, pregnancy in the time of examination, systemic disease that can affect eyes including diabetes, connective tissue disorders, atopy, allergy, and autoimmune disease.

Each participant underwent a complete ophthalmologic examination. Slit-lamp bio-microscopy, retinoscopy, fundoscopic examination, tomographic corneal assessment (tangential map of the corneas and the BAD-D value scores) via Pentacam HR, and corneal biomechanical evaluation utilizing Corvis ST (software version 1.6r2031) had been done during the same visit. Every measurement was taken from 9:00 AM to 2:00 PM to eliminate the probable effect of diurnal fluctuation [18].

Corvis ST (OCULUS Optikgeräte GmbH; Wetzlar, Germany) assesses the deformation of the cornea in response to an air puff, which can quantify the cornea’s stiffness and *in-vivo* viscoelastic properties [19]. This Scheimpflug imaging device collects parameters through first applanation (A1), highest concavity, and second applanation (A2) phases; A1 and A2 velocity (speed of corneal apex movement through first and second applanation), A1 and A2 length (length of the flattened cornea at first and second applanation), peak distance (distance between the two bending peaks of the cornea at the highest concavity state), concavity radius (the central corneal curvature radius at the highest concavity state), and deformation amplitude (DA, corneal apex displacement at the highest concavity) [20]. Furthermore, the following output parameters were also used: DA Ratio (the ratio between DA measured at the apex and 2 mm from the center of the cornea), Intraocular pressure (IOP), central corneal thickness (CCT), Integrated Radius (integrated area under the curvature of the inverse radius), Ambrósio relational thickness to the horizontal profile (ARTh, a parameter computed by the deviation of thinnest corneal thickness and Pachymetric Progression Index), stiffness parameter at the first applanation (SPA1, the resultant pressure of the first applanation computed as the adjusted pressure at A1 minus biomechanical corrected intraocular pressure divided by the deflection amplitude at A1), Corvis biomechanical index (CBI, integration of several biomechanical parameters), and Tomographic and biomechanical index (TBI, integration of corneal morphology and biomechanics) were included [5, 10, 20]. Biomechanical parameters were measured two times (10 min apart to eliminate possible measurement bias) by a single qualified technician under low light conditions [21, 22], and the average of measurements with “OK” quality-specifications was reported. The measurement technics and principles were described elsewhere [20].

IBM SPSS Statistics for Windows, version 26.0. (Armonk, NY: IBM Corp) and MedCalc for Windows, version 20.104 (MedCalc Software, Ostend, Belgium) were utilized for statistical analysis. The normality of the data distribution was evaluated via the Kolmogorov–Smirnov test. Data with normal distribution were presented as mean ± standard deviation (SD) and compared by Independent-Samples T test; otherwise, non-normally distributed data were presented as median [Minimum, Maximum] and were compared by Mann–Whitney U test. Depending on the data distribution, Spearman’s or Pearson’s correlation analysis was utilized to explore the association between CCT and Corvis ST-derived parameters. The analysis of covariance (ANCOVA) was operated to compare mean estimates parameters after adjusting the effect of IOP and CCT covariates on dynamic corneal response (DCR) parameters. The discriminatory performances of some cornea biomedical variables were assessed by analyzing the Receiver Operating Characteristic (ROC) curve. The area under the ROC curve (AUC) was calculated to distinguish the different variables between groups. The DeLong method [23] was used to assess the statistical significance of AUC pairwise comparisons. The Youden Index was utilized to determine the optimal threshold of the variables. *P*-value < 0.05 (two-tailed) was considered statistically significant.

## Results

**Fig. 1:**
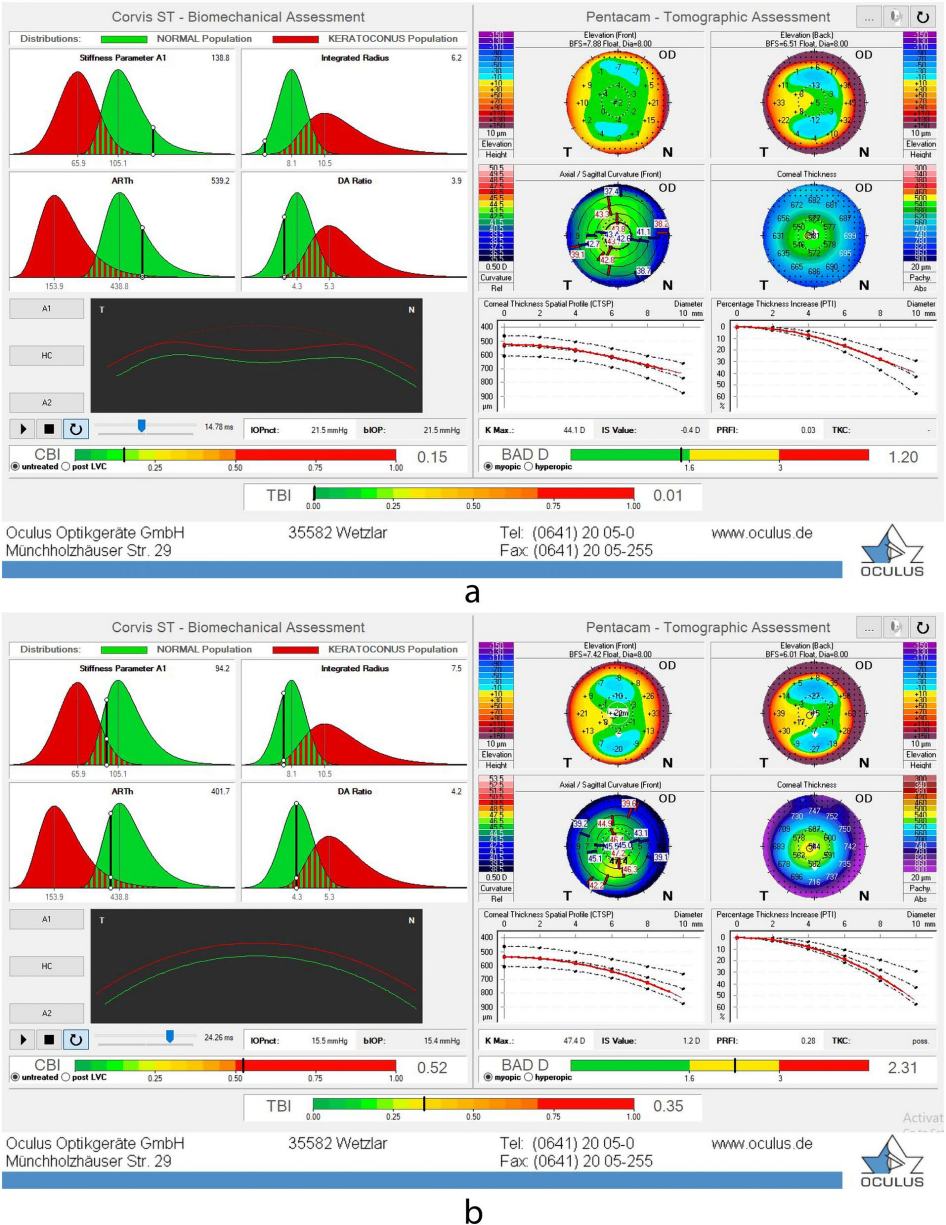
Biomechanical/Tomographic output of Corvis ST. **a** Normal participant, **b** Subclinical keratoconus case

Based on the inclusion and exclusion criteria, 73 keratoconus suspect corneas (from 73 participants) and 69 normal ones (from 69 participants) were involved. Figure 1 presents a normal cornea (a) and a keratoconus suspect one (b). No significant differences existed in the mean age of normal and subclinical keratoconus cases (Table 1). Also, the median IOP value among normal and keratoconus suspect eyes was mostly consistent (16 [11.50, 25], 16 [10.50, 27.50], respectively). However, the mean CCT value was significantly higher in the normal eyes (531.60 ± 31.07 μm) compared to the suspect ones (509.78 ± 31.31 μm) (*p*-value = 0.001). Compared to the suspect eyes, A1-length and A2-length were greater in normal eyes. Though, only the A2-length difference between groups was significant (*p*-value = 0.048). Moreover, normal eyes showed statistically significant higher rates for SPA-1 and ARTh. Conversely, DA ratio, CBI, and TBI presented significantly higher rates in keratoconus suspect eyes (all *p*-values < 0.05). After adjusting parameters for IOP and CCT, peak distance, ARTh, CBI, and TBI showed significant differences between the groups (Table 2).

**Table 1:** Characteristics of basic parameters and Corvis ST indices

*Parameters*	*Normal (n* = *69)*	*Subclinical Keratoconus (n* = *73)*	*P-value*
Age (years), median [min, max]	28 [19, 70]	31 [20, 58]	0.299
BAD-D (SDs), mean ± SD	1.03 ± 0.39	2.10 ± 0.34	**< 0.001**
IOP (mmHg), median [min, max]	16 [11.50, 25]	16 [10.50, 27.50]	0.509
CCT (μm), mean ± SD	531.60 ± 31.07	509.78 ± 31.31	**< 0.001**
A1 length (mm), median [min, max]	2.34 [1.71, 2.97]	2.26 [1.70, 2.75]	0.140
A2 length (mm), median [min, max]	1.51 [0.82, 2.28]	1.35 [0.84, 2.25]	**0.048**
A1 velocity (m/s), median [min, max]	0.14 [0.10, 0.19]	0.14 [0.10, 0.19]	0.217
A2 velocity (m/s), mean ± SD	-0.33 ± 0.03	-0.32 ± 0.04	0.425
Peak Distance (mm), median [min, max]	5 [4.47, 5.52]	4.96 [4.07, 5.98]	0.258
Radius (mm), mean ± SD	7.51 ± 0.91	7.27 ± 0.85	0.101
Deform Amplitude (mm), mean ± SD	1.06 ± 0.09	1.08 ± 0.11	0.428
DA ratio, median [min, max]	4.20 [3.10, 5.30]	4.30 [3.60, 5.70]	**0.006**
Integrated Radius (mm^−1^), mean ± SD	7.57 ± 1.07	7.90 ± 1.04	0.072
ARTh, median [min, max]	539.80 [377.80, 880.80]	440.10 [299.80, 832.40]	**< 0.001**
SPA1, mean ± SD	99.93 ± 17.18	93.04 ± 15.39	**0.013**
CBI, median [min, max]	0.36 [0.01, 0.95]	0.71 [0.01, 0.93]	**< 0.001**
TBI, median [min, max]	0.13 [0, 0.73]	0.57 [0.03, 1]	**< 0.001**

*BAD-D* Belin/Ambrósio enhanced ectasia total deviation value, *IOP *Intraocular pressure, *CCT* Central corneal thickness, *DA* Deformation amplitude, *ARTh* Ambrósio relational thickness to the horizontal profile, *SPA1* Stiffness parameter at the first applanation, *CBI* Corvis biomechanical index, *TBI* Tomographic and biomechanical index

**Table 2:** Corvis ST parameters in normal and subclinical keratoconus eyes

*Mean estimates value*	*Normal (n* = *69)*	*Subclinical Keratoconus (n* = *73)*	*P-value*
A1 length (mm)	2.30	2.25	0.331
A2 length (mm)	1.52	1.45	0.333
A1 velocity (m/s)	0.14	0.14	0.557
A2 velocity (m/s)	-0.33	-0.33	0.127
Peak Distance (mm)	5.02	4.95	**0.024**
Radius (mm)	7.46	7.33	0.406
Deform Amplitude (mm)	1.07	1.08	0.635
Deform Amplitude (DA) ratio	4.29	4.35	0.198
Integrated Radius (mm^−1^)	7.70	7.79	0.564
ARTh	545.38	451.94	**< 0.001**
SPA1	96.84	95.98	0.467
CBI	0.43	0.61	**< 0.001**
TBI	0.23	0.55	**< 0.001**

*ARTh* Ambrósio relational thickness to the horizontal profile, *SPA1* Stiffness parameter at the first applanation, *CBI* Corvis biomechanical index, *TBI* Tomographic and biomechanical index

**Fig. 2:**
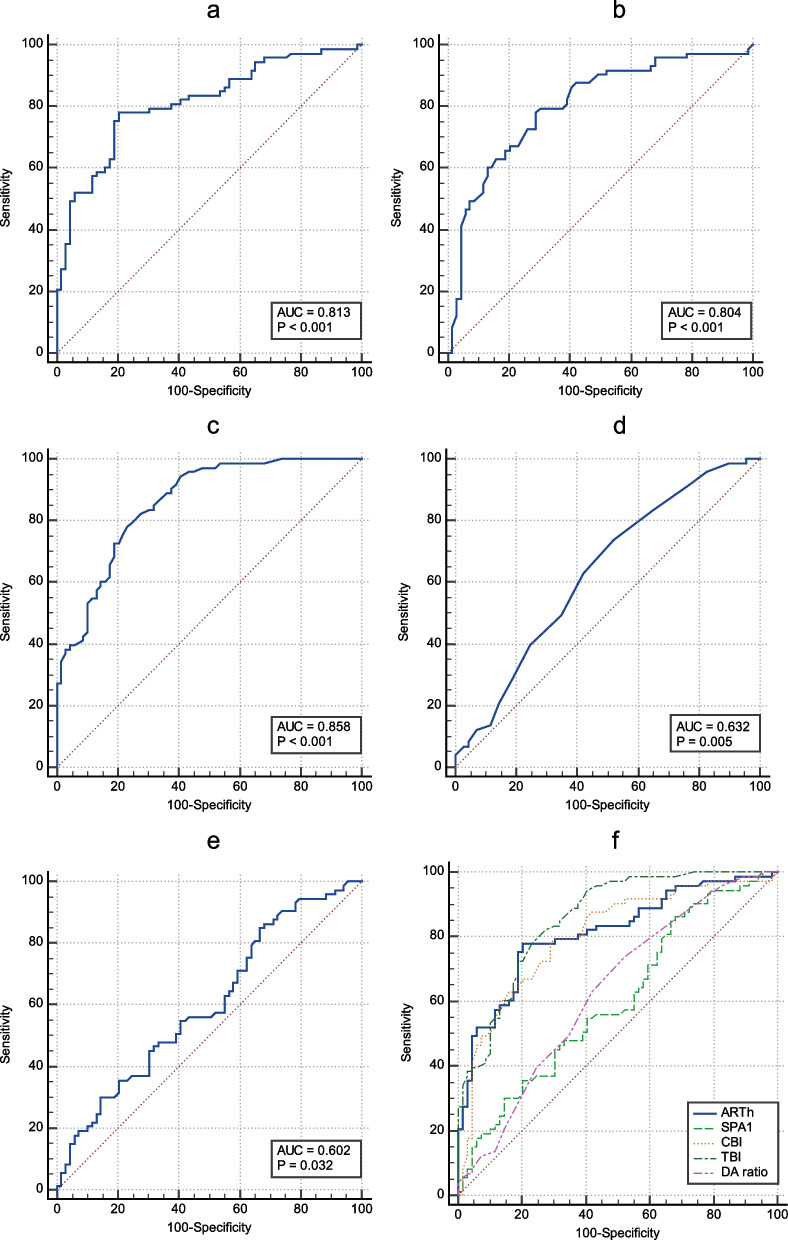
Receiver-Operating Characteristic (ROC) curves. **a** ARTh, **b** CBI, **c** TBI, **d** DA ratio, **e** SPA1, **f** Comparisons of the parameters

Receiver-operating characteristic (ROC) curves and AUC values of Corvis ST parameters with AUC value > 0.6 for detecting keratoconus suspect corneas, as well as the optimal threshold with the highest overall sensitivity and specificity, Youden index, and significance levels, are presented in Fig. 2 and Table 3. The area under the receiver operating characteristic (AUROC) curves for TBI, CBI, and ARTh revealed them as good indicators for distinguishing subclinical keratoconus eyes from normal ones. Moreover, SPA-1 and DA ratio showed fair discriminatory value. Figure 2(F) and Table 4 show the comparison of the ROC curves and AUROC curve values of the indices. Although CBI, TBI, and ARTh showed significantly higher AUROC values compared to SPA1 and DA ratio, no significant differences existed between CBI, TBI, and ARTh indices.

**Table 3:** Corvis ST parameters with AUC > 0.6 in distinguishing subclinical keratoconus corneas from normal ones

*Variable*	*AUC (95% CI)*	*Optimal threshold*	*Sensitivity*	*Specificity*	*Youden index*	*Significance level P (Area* = *0.5)*
ARTh	0.813 (0.740, 0.874)	≤ 488.1	78.08%	79.71%	0.5779	**< 0.0001**
CBI	0.804 (0.729, 0.866)	> 0.47	78.08%	71.01%	0.4910	**< 0.0001**
TBI	0.858 (0.790, 0.911)	> 0.33	78.08%	76.81%	0.5489	**< 0.0001**
DA ratio	0.632 (0.547, 0.711)	> 4.1	73.97%	47.83%	0.2180	**0.0048**
SPA1	0.602 (0.516, 0.683)	≤ 106.8	84.93%	33.33%	0.1826	**0.0321**

*AUC* Area under the curve, *CI* Confidence interval, *ARTh* Ambrósio relational thickness to the horizontal profile, *CBI* Corvis biomechanical index, *TBI* Tomographic and biomechanical index, *DA* Deformation amplitude, *SPA1* Stiffness parameter at the first applanation

**Table 4:** Pairwise comparison of area under the receiver operating characteristic curve values

*Differences between AUROC curve values*	*TBI*	*CBI*	*ARTh*	*SPA1*	*DA ratio*
TBI		0.0537	0.0446	**0.256**	**0.226**
CBI	0.0537		0.00913	**0.202**	**0.172**
ARTh	0.0446	0.00913		**0.212**	**0.181**
SPA1	**0.256**	**0.202**	**0.212**		0.0301
DA ratio	**0.226**	**0.172**	**0.181**	0.0301	

Bold ones were significant (*p*-value < 0.05)

*TBI* Tomographic and biomechanical index, *CBI* Corvis biomechanical index, *ARTh* Ambrósio relational thickness to the horizontal profile, *SPA1* Stiffness parameter at the first applanation, *DA* Deformation amplitude

**Fig. 3:**
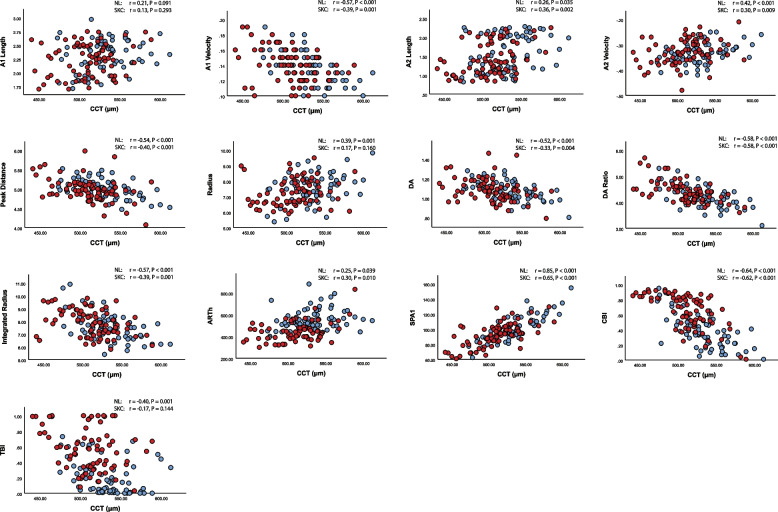
Scatter plot of Corvis ST parameters and CCT (Red circles: Subclinical keratoconus, Blue circles: Normal)

CCT were significantly (all *p*-values < 0.001) correlated (-0.685 ≤ r ≤ 0.760) with all the Corvis ST parameters. Furthermore, among the subclinical keratoconus eyes, the association analysis showed (Fig. 3) that CCT was significantly correlated with all parameters except radius at the highest concavity, A1-length, and TBI. On the other hand, among the normal eyes, CCT was significantly correlated with all the parameters except A1-length.

## Discussion

Keratoconus is the most prevalent ectatic corneal disorder, principally manifested as progressive thinning and steeping of the cornea [1, 24]. Due to the high acceptance rate of corneal refractive surgeries, early detection of this disorder has been a challenging topic and represents a substantial area of interest. Studies revealed that keratoconus corneas have a lower deformation resistance and higher deformation amplitude after the air-puff force than normal ones. Thus, the theory of biomechanics alteration as the pathogenesis of keratoconus was developed, and in-vivo biomechanical parameters have been evaluated as the primary diagnostic criteria in keratoconus suspect corneas [20, 25].

Consistent with previous studies [19, 26], DA ratio, ARTh, SPA1, CBI, and TBI showed significant differences between normal and subclinical keratoconus groups. Apart from A2-length, which in theory we expect higher values in the suspect corneas and conversely presented lower values compared to normal eyes, arrays of the parameters were consistent with the fact that subclinical keratoconus corneas have decreased viscoelastic and stiffness and increased distensibility [27]. Nevertheless, the reported results of A2-length were in line with other studies [28]. Following IOP and CCT adjustment, A2-length, SPA1, and DA ratio lost their significant differences. However, a recent study among the Chinese population [19] stated that after adjusting for IOP and CCT, DA ratio, and ARTh lost their significant divergence between the normal and subclinical groups, but SPA1 and CBI remained significantly different between the groups. The mean estimated value of the peak distance (PD) at the highest concavity presented significantly higher in the normal group after the adjustment, which is contradictory to other studies that exhibited lower peak distance rate compared to subclinical keratoconus eyes [27].

Regarding the association between CCT and the biomechanical parameters, the outcomes of studies vary. A retrospective study on 184 normal eyes and 28 subclinical keratoconus eyes stated significant (all *p*-values < 0.001) correlations of CCT with DA, A1-length, A2-velocity, and radius at the highest concavity, and no significant correlations (*p*-value > 0.05) with A2-length, A1-velocity and peak distance [29]. A cross-sectional clinical study among South Asian population confirmed significant correlation of CCT with A1-velocity (*r* = -0.299), DA ratio (*r* = -0.554), ARTh (*r* = 0.453), SPA1 (*r* = 0.649), CBI (*r* = -0.366), and TBI (*r* = -0.239), among the normal eyes [11]. Furthermore, a recent study on Chinese population revealed significant (all *p*-value < 0.001) correlation of CCT with following Corvis ST parameters among both normal and subclinical keratoconus eyes; Integrated radius (r_NL_ = -0.41, r_SKN_ = -0.43), DA ratio (r_NL_ = -0.56, r_SKN_ = -0.49), SPA1 (r_NL_ = 0.63, r_SKN_ = 0.72), CBI (r_NL_ = -0.51, r_SKN_ = -0.4). They also stated no significant correlation between CCT and ARTh among both groups [19].

On the subject of the discriminatory value of the Corvis ST parameters, studies reported varied AUROC curve values and optimum threshold rates. Compared to the results of our study, Heidari et al. [26], in a study with quite similar inclusion criteria (subclinical keratoconus defined without the necessity of clinical keratoconus diagnosis in the other eye), reported higher AUROC curve values for SPA1 (0.779) and DA ratio (0.742), and lower AUROC curve values for ARTh (0.718), CBI (0.758), and TBI (0.828) in differentiating subclinical keratoconus from normal eyes. However, due to the use of BAD-D value in the subclinical keratoconus inclusion criteria of our study, the TBI accuracy should be discussed with caution. A novel study on 47 keratoconus suspect eyes, defined as frank keratoconus fellow eyes with normal or borderline topographically/tomographically features, described TBI (AUROC: 0.946), SPA1 (0.833), and CBI (0.822) as valuable discriminators of topographically/tomographically borderline fellow eyes from normal ones [30]. Also, Ambrosio et al. [5] stated high discriminatory value of TBI (AUROC: 0.985, cut-off: 0.29, sensitivity: 0.904, specificity: 0.960), and CBI (AUROC: 0.822, cut-off: 0.07, sensitivity: 0.681, specificity: 0.823) in distinguishing very asymmetric ectasia with normal topography (VAE-NT) eyes from normal ones. Another study on 79 normal and 27 VAE-NT eyes among the Chinese population confirmed the discriminatory values of TBI (AUROC: 0.928, cut-off: 0.38, Youden index: 0.753), and CBI (AUROC: 0.860, cut-off: 0.27, Youden index: 0.642) for distinguishing early keratoconus from normal eyes [21]. Other studies that evaluated the TBI discriminatory value in distinguishing VAE-NT from normal eyes reported AUROC values from 0.751 with a cut-off value of 0.259 [31] to 0.925 with a cut-off value of 0.16 [32].

Kataria et al. [11], in a cross-sectional clinical study among South Asian participants, reported a good discriminatory value of SPA1 (AUROC: 0.762, cut-off: 93.74, sensitivity: 66, specificity: 83) and CBI (AUROC: 0.775, cut-off: 0.01, sensitivity: 68, specificity: 77) in distinguishing VAE-NT from normal eyes. Compared to our results, Ren et al. conducted a study among Chinese population and reported lower AUROC curve values for CBI (AUROC: 0.703, cut-off: > 0.05, Youden index: 0.38) and ARTh (AUROC: 0.618, cut-off: ≤ 434.02, Youden index: 0.3), and higher AUROC curve values for DA ratio (AUROC: 0.684, cut-off: > 4.47, Youden index: 0.33), and SPA1 (AUROC: 0.753, cut-off: ≤ 107.3, Youden index: 0.4) in distinguishing subclinical keratoconus from normal eyes [19].

Ethnic diversity and discrepancy in the utilized inclusion criteria among the studies could be the reason for the resulting variations in the reported AUROC curve values and optimum cut-off values. Considering that most of the previous studies evaluated the non-Caucasian population and included the presence of clinical keratoconus in the fellow eye as one of the subclinical keratoconus inclusion criteria [33], the current study aimed to evaluate the diagnostic value of biomechanical parameters in subclinical keratoconus (normal appearing corneas with suspicious topographic/tomographic features) cases. The small sample size and the lack of follow-up for subclinical keratoconus cases are the major limitations of our study.

## Conclusions

TBI, CBI, and ARTh parameters presented good discriminatory values in distinguishing subclinical keratoconus eyes from normal ones.

## Data Availability

The datasets used and analyzed during the current study are available from the corresponding author on reasonable request.

## Abbreviations

Corvis ST: Corneal Visualization Scheimpflug Technology
SKC: Subclinical keratoconus
DA: Deformation Amplitude
TBI: Tomographic Biomechanical Index
CBI: Corvis Biomechanical Index
ARTh: Ambrósio Relational Thickness to the Horizontal profile
SPA1: Stiffness Parameter at the first Applanation
BAD-D: Belin/Ambrósio enhanced ectasia total deviation
A1: First applanation
A2: Second applanation
IOP: Intraocular pressure
CCT: Central corneal thickness
SD: Standard deviation
ANCOVA: Analysis of covariance
DCR: Dynamic corneal response
ROC: Receiver Operating Characteristic
AUC: Area under curve
AUROC: Area under the receiver operating characteristic
